# Bowman’s capsule rupture and its clinical significance in patients with anti-glomerular basement membrane disease

**DOI:** 10.3389/fimmu.2025.1655319

**Published:** 2025-12-04

**Authors:** Huang Kuang, Cai-xia Lin, Xiao-juan Yu, Zhao Cui, Ming-hui Zhao, Xiao-yu Jia

**Affiliations:** 1 Renal Division, Peking University First Hospital, Beijing, China; 2 Institute of Nephrology, Peking University, Beijing, China; 3 Key Laboratory of Renal Disease, Ministry of Health of China, Beijing, China; 4 Key Laboratory of CKD Prevention and Treatment, Ministry of Education of China, Beijing, China

**Keywords:** anti-glomerular basement membrane disease, crescentic glomerulonephritis, Bowman’s capsule rupture, end-stage kidney disease, prognosis

## Abstract

**Background:**

Anti-glomerular basement membrane (GBM) disease represents the most severe form of crescentic glomerulonephritis. Previous studies demonstrated that Bowman’s capsule rupture contributed to the progression of crescentic glomerulonephritis. However, its role in anti-GBM disease remains unclear. The aim of this study was to investigate the prevalence and severity of Bowman’s capsule rupture in patients with anti-GBM disease and its clinical associations.

**Methods:**

A total of 72 patients diagnosed with biopsy-proven anti-GBM disease with complete clinical and pathologic data were retrospectively enrolled.

**Results:**

Extensive Bowman’s capsule rupture occurred in 70 patients (97.2%) with a median percentage of 52.8% of all glomeruli on each kidney biopsy. The percentage of Bowman’s capsule rupture showed a strong association with kidney injuries (incidence of oligoanuria, eGFR, and serum creatinine on diagnosis; *P* < 0.001) and the levels of anti-GBM antibody (*P* = 0.013). Histologically, Bowman’s capsule rupture percentage was positively correlated with crescent percentage (*P* = 0.001) and increased proportion of cellular–fibrous crescents specifically (*P* = 0.047). The Kaplan–Meier analysis revealed significantly divergent outcomes in kidney survival (*P* = 0.006) and kidney recovery (*P* = 0.016) when the patients were divided into different groups according to the percentage of Bowman’s capsule rupture. The incorporation of Bowman’s capsule rupture into two proposed prediction models of risk stratification tool and renal risk score could improve their prognostic performance.

**Conclusions:**

Bowman’s capsule rupture serves as both a distinct histopathological feature and a critical determinant of kidney injury in anti-GBM disease. More importantly, as a simple, standalone parameter, it demonstrates a robust predictive value for kidney outcomes in patients with anti-GBM disease.

## Introduction

Anti-glomerular basement membrane (GBM) disease is a rare autoimmune kidney disease characterized by the presence of pathogenic autoantibodies against the GBM components, causing rapidly progressive glomerulonephritis with diffuse crescent formation on kidney biopsies (1). Although the utilization of aggressive immunosuppressive therapies leads to remarkable improvements in patient survival, kidney outcomes remain suboptimal. A substantial proportion of patients (60%–80%) progress to end-stage kidney disease (ESKD) within the first year of follow-up, subsequently requiring renal replacement therapy (RRT) (2). Therefore, identifying clinical or pathological indicators to predict kidney prognosis would be useful for clinicians to make individualized treatment regimens (3–5).

Bowman’s capsule is the outer epithelial wall of the glomerular corpuscle and serves as a physical barrier to protect the glomerulus, which consists of a parietal layer composed of a single layer of flat partial epithelial cells (PECs). The proliferating PEC, the core component of Bowman’s capsule, is a pathological hallmark of crescentic glomerulonephritis (6–9). In recent years, accumulating evidence underscores the critical contribution of Bowman’s capsule rupture in the development of crescentic glomerulonephritis (10–12). We previously revealed that the prevalence of glomerular infiltrating CD8^+^ T cells was correlated with the percentage of Bowman’s capsule rupture in patients with anti-GBM disease (11). In addition, Chen et al. further elucidated that the peri-glomerular pathogenic CD8^+^ T cells attacked Bowman’s capsule, inducing its rupture and thereby exacerbating experimental anti-GBM nephritis (12). These findings together emphasize the essential involvement of Bowman’s capsule rupture in the pathogenesis of anti-GBM disease. However, the clinical association between the extent of Bowman’s capsule rupture on kidney biopsies and kidney prognosis in anti-GBM disease has not been previously studied. Therefore, in the present study, we aim to investigate the landscape of Bowman’s capsule rupture and its clinical significance in a large cohort of patients with biopsy-proven anti-GBM disease.

## Materials and methods

### Patients

A total of 72 patients diagnosed with biopsy-proven anti-GBM disease in Peking University First Hospital between 2005 and 2022 were enrolled. The diagnosis of anti-GBM diseases was based on serum detection of anti-GBM antibodies and kidney biopsy showing linear IgG fluorescence along the GBM. Anti-GBM antibodies were detected by commercial ELISA kits (Euroimmun, Luebeck, Germany). Patients with following criteria were excluded: (a) combined with other forms of crescentic glomerulonephritis, such as antineutrophil cytoplasm antibodies (ANCA)-associated vasculitis and lupus nephritis, (b) inadequate number of glomeruli on biopsy (<10), (c) other causes that resulted in linear IgG deposition, such as diabetes and fibrillary glomerulonephritis, and (d) loss of biopsy slides or incomplete clinical–pathological data. This study was approved by the Ethics Committee of Peking University First Hospital and in accordance with the Declaration of Helsinki.

### Clinical parameters and kidney histopathology

ESKD is defined as the maintenance of kidney replacement therapy (KRT) for at least 12 weeks and up to the last follow-up. Kidney recovery is defined as independence from RRT for at least 12 weeks during follow-up. At least eight consecutive levels were performed to assess different pathological indicators with various stains, including periodic acid–Schiff (PAS), hematoxylin and eosin (H&E), Masson, and dual PAS + Masson. For the evaluation of Bowman’s capsule rupture, kidney tissues primarily stained with periodic acid–Schiff and Masson were independently evaluated by two pathologists using light microscopy. Normal glomeruli are defined as glomeruli that did not exhibit glomerulosclerosis, crescents, or fibrinoid necrosis. Renal risk score (RRS) was calculated as described previously (4). Bowman’s capsule rupture was defined as a discontinuity in the wall of Bowman’s capsule on kidney biopsy sections. When a fibrous crescent occurred within the glomerulus in which collagen fibers may mimic a capsule or form a pseudo-capsule, the glomerulus was only defined as having Bowman’s capsule rupture if a suspected discontinuity in the capsule was accompanied by the presence of peri-glomerular inflammatory cell infiltration (as identified on H&E staining). The Bowman’s capsule rupture percentage was calculated as a fraction of the affected glomeruli divided by the total number of glomeruli on each kidney biopsy.

### Statistics

Data are shown as the median and interquartile range (IQR) for continuous variables and number (%) for qualitative variables. The regression tree analysis was performed using the R package *rpart* (version 4.1.19; R Core Team, Vienna, Austria) to determine the cutoff values of Bowman’s capsule rupture percentage for categorizing patients into different groups. The Cochran–Armitage trend test was performed to compare the baseline data among groups. Factors predictive of kidney outcomes were evaluated by using Kaplan–Meier analysis, Cox proportional hazard model with discrimination capability (assessed by Harrell’s *C* statistic), and receiver operating characteristic (ROC) curves. *P*-value <0.05 was considered significant. All statistical analyses were performed using R v4.0.5.

## Results

### Characteristics of the study participants

**Figure 1:**
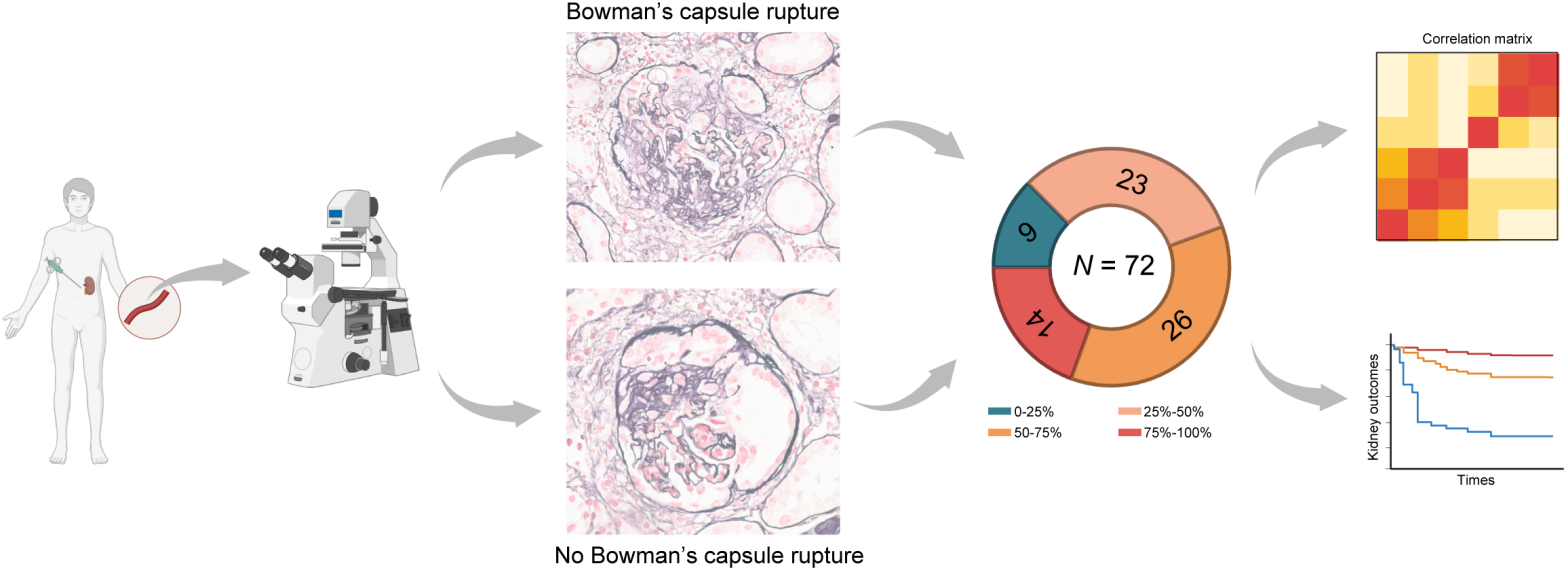
Workflow and clinical outcomes of Bowman’s capsule rupture in patients with anti-GBM diseases. A total of 72 patients diagnosed with anti-GBM diseases who underwent kidney biopsies were enrolled. The severity of Bowman’s capsule rupture was evaluated using light microscopy. The proportion of individuals with varying Bowman’s capsule rupture percentage on the biopsies is shown in the pie chart. Correlation and prognostic analyses were performed between Bowman’s capsule rupture percentage and parameters.

A total of 72 patients with biopsy-confirmed anti-GBM disease were included in our study (**Figure 1**). The cohort demonstrated a balanced gender distribution (51.4% female and 48.6% male) and a median age of 43.5 years (IQR: 33.0-55.8) at diagnosis. The median serum creatinine on diagnosis was 613.8 μmol/L, and the median level of anti-GBM antibody was 156.00 RU/mL. A total of 49 (68.1%) patients received RRT at presentation. All patients (100%) received glucocorticoids, and 58 (80.6%) patients received cyclophosphamide.

During follow-up, 14/49 (28.6%) patients among those requiring RRT at presentation achieved kidney recovery, 40/72 (55.6%) patients progressed to ESKD, and one death (1.4%) occurred among all patients (**Table 1**).

### Correlation between Bowman’s capsule rupture and the clinical features of anti-GBM patients

The histopathological evaluation demonstrated that the presence of Bowman’s capsule rupture was observed in 97.2% (70/72) of all patients, with a median percentage of 52.8% (42.3%, 71.4%) among all glomeruli. To evaluate the association of the percentage of Bowman’s capsule rupture among all glomeruli in patients with anti-GBM disease with kidney prognosis, the cutoff values for Bowman’s capsule rupture percentage were established using a regression tree analysis according to the status of kidney survival (**Supplementary Figure S1**). Three applicable cutoff values for Bowman’s capsule rupture percentage at 25%, 50%, and 75% were identified and thus categorized the patients into four groups (group 1: 0%–25%, *n* = 9; group 2: 25%–50%, *n* = 23; group 3: 50%–75%, *n* = 26; and group 4: 75%–100%, *n* = 14). Patients in higher Bowman’s capsule rupture categories had increasingly higher rates of oligoanuria incidence (*P* < 0.001), accompanied by marked declines in kidney function including serum creatinine (*P* < 0.001) on diagnosis and estimated glomerular filtration rate (eGFR, *P* < 0.001; **Table 1**). Patients in higher Bowman’s capsule rupture categories, particularly groups 3 and 4, had increased levels of anti-GBM antibody (*P* = 0.013). The median proportion of normal glomeruli decreased significantly from 50.0% in group 1 to 0.0% in group 4 (*P* = 0.001), along with an increased median percentage of cellular–fibrous crescents from 17.1% to 48.8% (*P* = 0.047). More importantly, the rates of RRT initiation increased from 22.2% in group 1 to 92.9% in group 4 (*P* = 0.001), while the progression to ESKD increased from 22.2% to 85.7% (*P* = 0.012). Notably, the rates of kidney recovery were inversely correlated with Bowman’s capsule rupture percentage, declining from 50.0% in group 1 to 7.7% in group 4.

**Table 1:** Baseline characteristics and comparison of patients in groups according to Bowman’s capsule rupture percentage.

Characteristics	Total (*n* = 72)	Group 1 (0%–25%) (*n* = 9)	Group 2 (25%–50%) (*n* = 23)	Group 3 (50%–75%) (*n* = 26)	Group 4 (75%–100%) (*n* = 14)	*P*-value
Age, years	43.5 (33.0, 55.8)	43.0 (32.0, 48.0)	46.0 (34.5, 55.5)	43.5 (32.5, 57.3)	40.0 (34.5, 51.8)	0.777
Female (%)	37 (51.4)	3 (33.3)	10 (43.5)	12 (46.2)	12 (85.7)	**0.034**
Prodromal infection (%)	30 (41.7)	5 (55.6)	11 (47.8)	6 (23.1)	8 (57.1)	0.105
Hydrocarbon (%)	8 (14.0)	1 (14.3)	3 (16.7)	3 (15.0)	1 (8.3)	0.931
Hemoptysis (%)	14 (19.4)	3 (33.3)	5 (21.7)	3 (11.5)	3 (21.4)	0.521
Oligoanuri	23 (31.9)	0 (0.0)	4 (17.4)	8 (30.8)	11 (78.6)	**<0.001**
eGFR (mL/min/1.73m^2)	7.2 (4.9, 18.9)	46.5 (16.4, 64.9)	11.2 (5.2, 20.8)	6.3 (5.1, 10.6)	3.6 (2.4, 6.3)	**<0.001**
Serum creatinine (μmol/L)	613.8 (301.2, 882.5)	124.0 (110.0, 391.0)	493.0 (272.3, 785.1)	677.2 (431.50, 842.2)	1051.5 (678.0, 1549.0)	**<0.001**
Anti-GBM Ab (RU/mL)	156.0 (74.5, 200.0)	90.5 (59.0, 174.5)	88.0 (65.5, 178.0)	157.0 (55.3, 200.0)	200.0 (200.0, 200.0)	**0.013**
Bowman’s capsule rupture (%)	52.8 (42.3, 71.4)	10.7 (10.0, 20.0)	43.8 (34.0, 47.5)	65.9 (54.8, 69.8)	86.2 (84.7, 93.7)	**<0.001**
Normal glomeruli (%)	8.7 (0.0, 29.6)	50.0 (27.2, 66.7)	11.7 (6.3, 36.7)	7.7 (0.0, 22.0)	0.0 (0.0, 6.9)	**0.001**
Crescents (%)	85.4 (57.1, 92.9)	39.0 (32.0, 57.8)	78.6 (53.0, 90.1)	85. (68.0, 92.2)	93.9 (90.3, 100.0)	**0.001**
Cellular crescent (%)	33.4 (16.7, 52.9)	25.0 (7.7, 50.0)	34.6 (14.4, 51.5)	27.8 (17.5, 50.7)	40.3 (28.3, 79.4)	0.667
Cellular-fibrous crescent (%)	35.0 (16.4, 58.7)	17.1 (0.0, 26.3)	32.0 (19.9, 48.5)	46.7 (27.3, 60.7)	48.83 (10.0, 74.7)	**0.047**
Fibrous crescent (%)	0.0 (0.0, 7.0)	0.0 (0.0, 5.3)	0.0 (0.0, 3.9)	0.0 (0.0, 6.7)	0.0 (0.0, 8.0)	0.943
Initial need for RRT (%)	49 (68.1)	2 (22.2)	13 (56.5)	21 (80.8)	13 (92.9)	**0.001**
Glucocorticoids (%)	72 (100.0)	9 (100.0)	23 (100.0)	26 (100.0)	14 (100.0)	-
Cyclophosphamide (%)	58 (80.6)	9 (100.0)	16 (69.6)	21 (80.8)	12 (85.7)	0.242
Plasma exchange (%)	65 (90.3)	8 (88.9)	20 (87.0)	23 (88.5)	14 (100.0)	0.590
Kidney recovery (%)	14 (28.6)	1 (50.0)	6 (46.2)	6 (28.6)	1 (7.7)	0.158
ESKD (%)	40 (55.6)	2 (22.2)	10 (43.5)	16 (61.5)	12 (85.7)	**0.012**
Mortality (%)	1 (1.4)	0 (0.0)	0 (0.0)	0 (0.0)	1 (7.1)	0.248
Follow-up, months	24.0 (5.5, 36.9)	36.0 (24.0, 44.3)	16.0 (5.4, 36.3)	25.0 (3.5, 37.1)	21.3 (12.1, 31.2)	0.570

Number (%) and median values (interquartile range) are shown. Statistically significant differences are reported with a bold *P*-value.

eGFR, estimated glomerular filtration rate; GBM, glomerular basement membrane; RRT, renal replacement therapy; ESKD, end-stage kidney disease.

**Figure 2:**
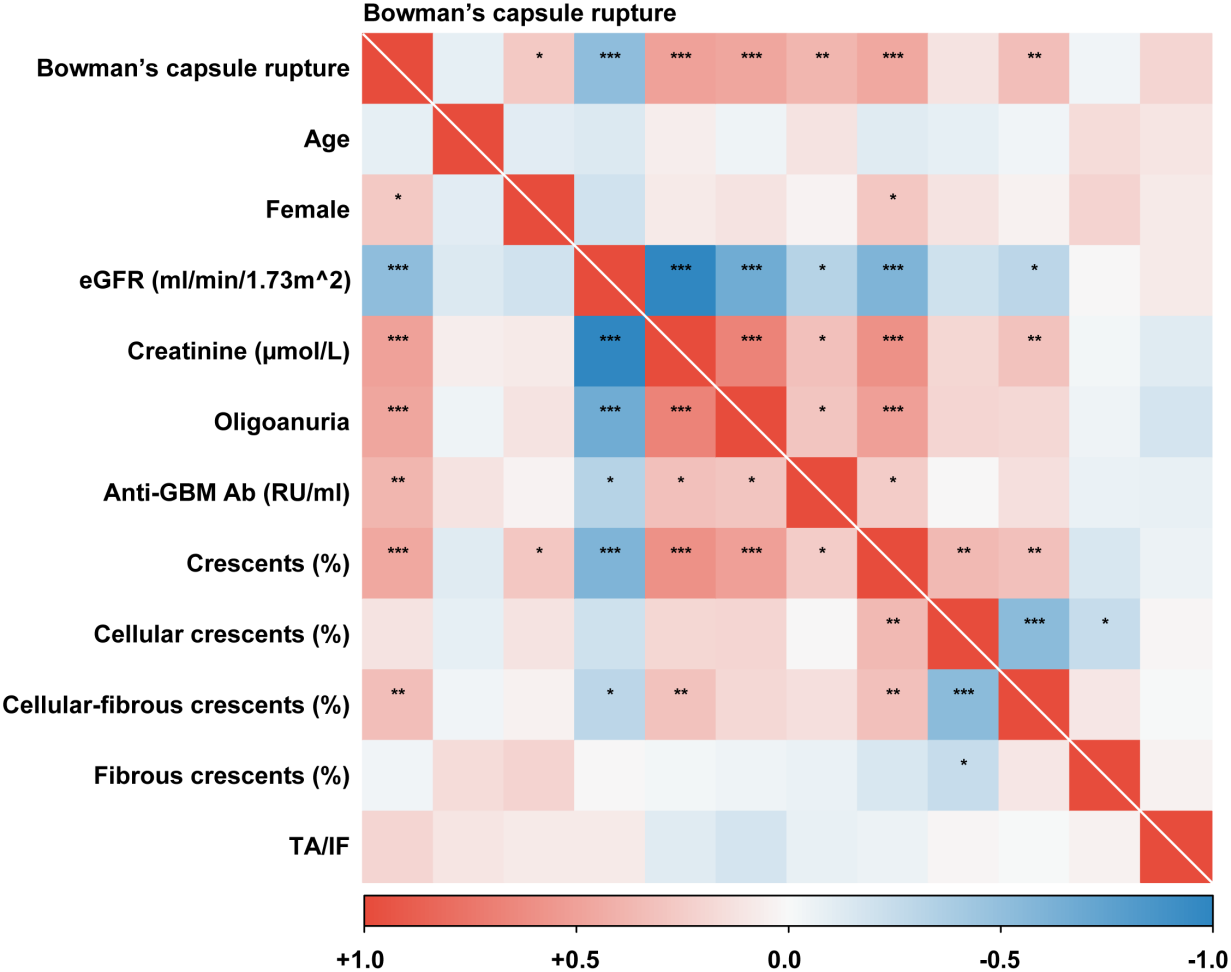
Bowman’s capsule rupture is associated with clinical and pathological characteristics. An association between Bowman’s capsule rupture and parameters in patients with GBM disease is shown by a heatmap reflecting the *P*-value of Spearman’s analysis. Bowman’s capsule rupture was defined as a continuous variable here. eGFR, estimated glomerular filtration rate; Anti-GBM Ab, anti-glomerular basement membrane antibody; TA/IF, tubular atrophy/interstitial fibrosis. *P < 0.05, **P < 0.01, ***P < 0.001.

As demonstrated in **Figure 2** by correlation analysis, Bowman’s capsule rupture percentage exhibited a strong positive correlation with serum creatinine on diagnosis (*r* = 0.500, *P* < 0.001), oligoanuria incidence (*r* = 0.478, *P* < 0.001), levels of anti-GBM antibody (*r* = 0.386, *P* = 0.003), crescents (*r* = 0.451, *P* < 0.001), and cellular–fibrous crescents (*r* = 0.330, *P* = 0.004). Conversely, a significant inverse correlation was observed between Bowman’s capsule rupture percentage and eGFR (*r* = -0.510, *P* < 0.001).

### Bowman’s capsule rupture and kidney outcomes of anti-GBM patients

**Figure 3:**
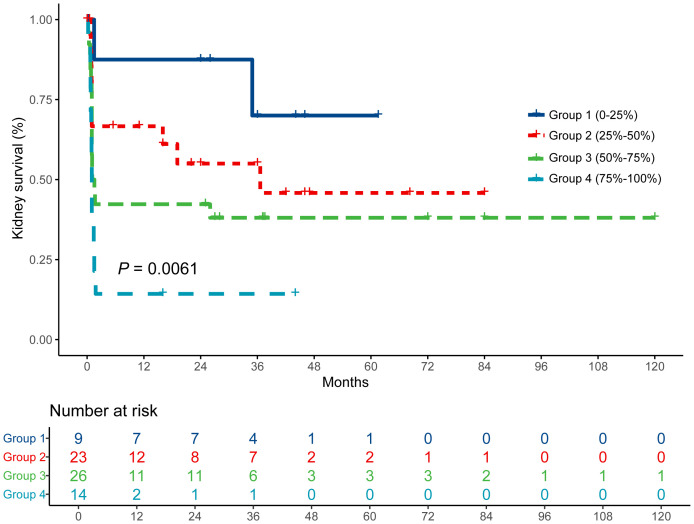
Kidney survival curves of the cumulative incidence of end-stage kidney disease. The patients were divided into four groups according to the percentage of Bowman’s capsule rupture on biopsies. A comparison of survival curves was performed with log-rank test.

A Kaplan–Meier analysis of kidney survival across the four-tiered Bowman’s capsule rupture severity classification revealed progressively worsening prognosis corresponding to increased Bowman’s capsule rupture severity (*P* = 0.006; **Figure 3**). Temporal survival patterns revealed early clinical differentiation: at 12, 24, and 36 months, the cumulative proportion of kidney survival was 87.5%, 87.5%, and 70.0% in group 1; 66.7%, 55.0%, and 55.0% in group 2; 42.3%, 42.3% and 38.1% in group 3; and 14.3%, 14.3%, and 14.3% in group 4. Patients in group 4 had the highest proportion of ESKD during follow-up: 12 of 14 patients progressed into ESKD, and 13 of 14 patients required RRT, with only one patient who had initial kidney recovery.

**Figure 4:**
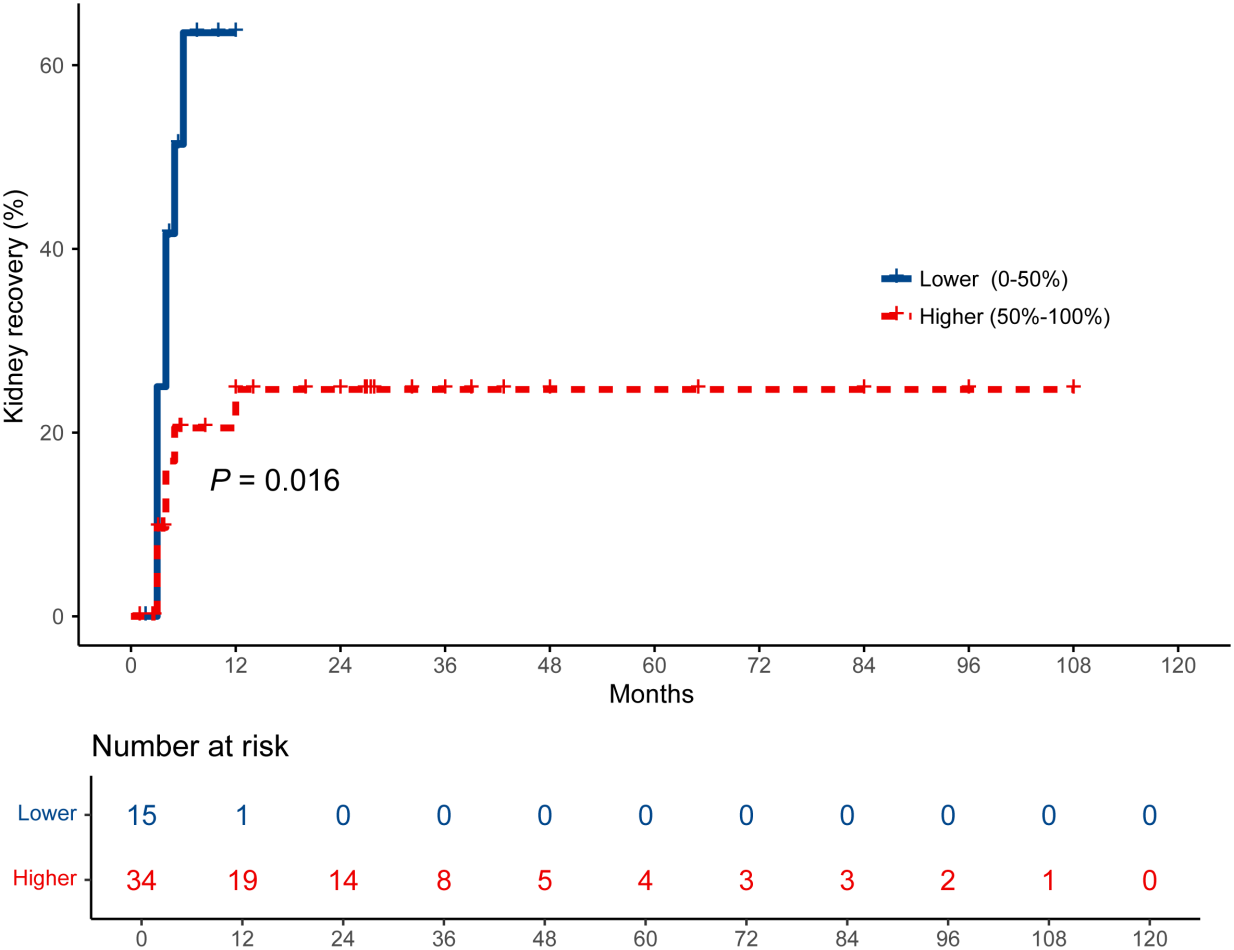
Kidney recovery curves of patients receiving initial renal replacement therapy. The patients were divided into two groups according to the percentage of Bowman’s capsule rupture on biopsies. A comparison of survival curves was performed with log-rank test.

To evaluate the prognostic significance of Bowman’s capsule rupture percentage in predicting kidney recovery, a regression tree analysis was performed to identify optimal cutoff values (**Supplementary Figure S2**). Stratification by a 50% Bowman’s capsule rupture percentage threshold significantly predicted kidney recovery, with the lower Bowman’s capsule rupture group (≤50%) showing superior recovery rates compared to the higher Bowman’s capsule rupture group (>50%). A survival analysis revealed statistically significant disparities in the chance of kidney recovery for both groups (*P* = 0.016; **Figure 4**). The cumulative proportion of kidney recovery at 3, 6, and 12 months was 25.0%, 63.5%, and 63.5% in lower Bowman’s capsule rupture group and 9.7%, 20.5%, and 24.7% in higher Bowman’s capsule rupture group.

### Bowman’s capsule rupture improves the prognostic performance of clinical prediction tools

**Figure 5:**
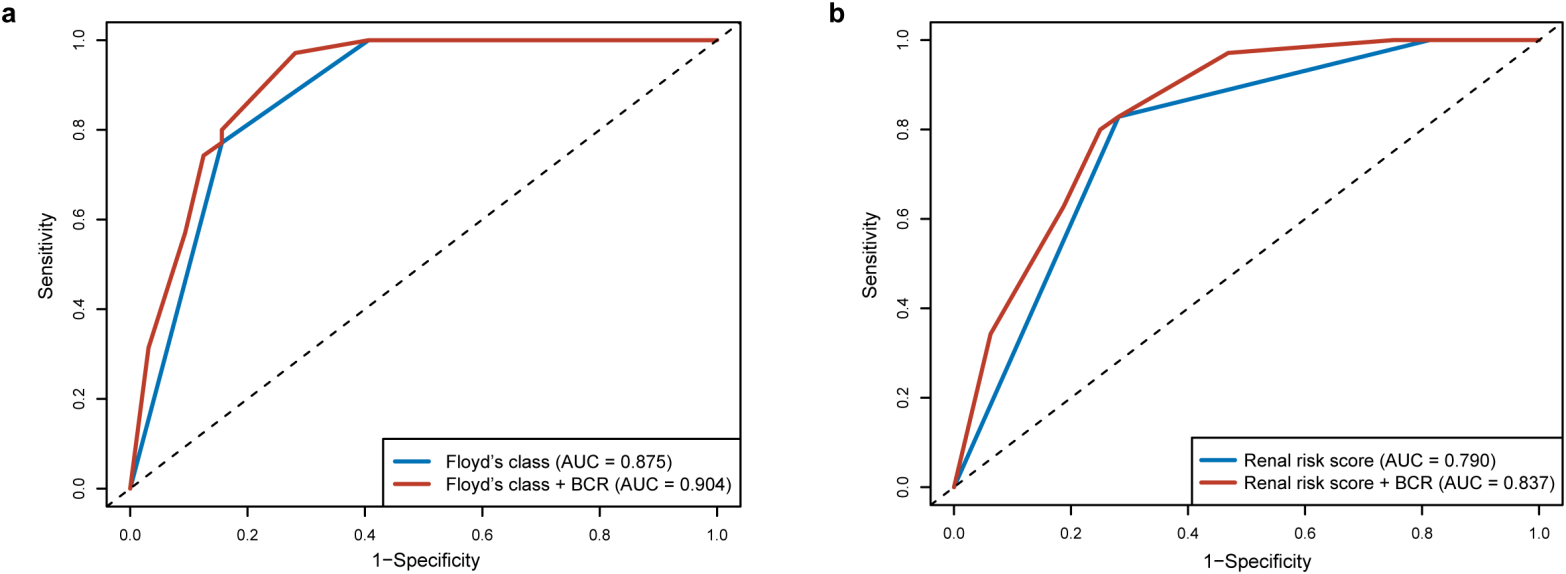
Comparison of prognostic performance for the severity of Bowman’s capsule rupture (BCR) and prediction tools. **(a)** Comparison of receiver operating characteristic (ROC) curves between Floyd’s class alone and Floyd’s class with Bowman’s capsule rupture. **(b)** Comparison of ROC curves between renal risk score alone and renal risk score with Bowman’s capsule rupture. Bowman’s capsule rupture was divided into degrees 1–4 (degree 1: 0%–25%, degree 2: 25%–50%, degree 3: 50%–75%, and degree 4, 75%–100%).

A risk stratification tool was recently established by Floyd et al. by stratifying the patients according to the percentage of normal glomeruli and the initial need for RRT (3). The tool was further validated to predict kidney survival in patients with anti-GBM disease in our previous studies (4). Besides that, a RRS model in ANCA-associated vasculitis was also extended to anti-GBM disease with favorable predictive performance. Among the histological predictors included in the current study, Bowman’s capsule rupture was significantly associated with kidney survival in the univariate analysis (*P* < 0.001 and *C* = 0.660, **Table 2**). The addition of Bowman’s capsule rupture to the risk stratification tool (Floyd’s class, *P* < 0.001 and *C* = 0.787) or the RRS tool alone (*P* < 0.001 and *C* = 0.720) improved the prognostic performance of the models (*C* = 0.802 and *C* = 0.746, respectively). The ROC analysis also demonstrated improved discriminatory performance for the combined models (Floyd’s class + Bowman’s capsule rupture: AUC = 0.904; RRS + Bowman’s capsule rupture: AUC = 0.837) compared to their individual counterparts (Floyd’s class: AUC = 0.875; RRS: AUC = 0.790) at 12-month follow-up although statistical significance was not achieved (*P* = 0.115 and *P* = 0.069, respectively; **Figures 5a, b**).

**Table 2:** Prognostic performance of pathological indicators for kidney survival.

Predictor	C-index	HR (95% CI)	*P*-value
Univariate analysis			
Active lesions			
Cellular crescents	0.574	1.010 (1.000 to 1.021)	0.067
Fibrocellular crescents	0.596	1.013 (1.001 to 1.025)	**0.031**
Fibrinoid necrosis	0.533	0.998 (0.982 to 1.015)	0.840
Bowman’s capsule rupture	0.660	1.835 (1.276 to 2.640)	**<0.001**
Chronic lesions			
Fibrous crescent	0.540	0.978 (0.939 to 1.020)	0.262
Glomerulosclerosis	0.581	0.647 (0.341 to 1.228)	0.183
Tubular atrophy/interstitial fibrosis	0.491	0.967 (0.545 to 1.884)	0.967
Multivariate analysis			
Floyd’s class only	0.787	3.512 (2.144 to 5.753)	**<0.001**
Floyd’s class + Bowman’s capsule rupture	0.802		
Renal risk score only	0.720	4.997 (2.456 to 10.170)	**<0.001**
Renal risk score + Bowman’s capsule rupture	0.746		

Statistically significant differences are reported with a bold *P*-value.

C-index, Harrell’s concordance statistic; HR, hazard ratio; 95% CI, 95% confidence interval; BCR, Bowman’s capsule rupture.

## Discussion

In this study, we found that the presence of extensive Bowman’s capsule rupture was closely associated with clinical and histopathological parameters of kidney injuries in patients with anti-GBM disease, indicating a possible role of Bowman’s capsule rupture in its pathogenesis. A key finding of our study was that Bowman’s capsule rupture may serve as a valuable prognostic indicator for kidney outcomes and enhance the performance of existing prognostic tools in patients with anti-GBM disease. Bowman’s capsule rupture is widely regarded as a pathological signature of severe glomerular injury. In the current study, we revealed that a majority of patients (97.2%) with anti-GBM disease showed a feature of Bowman’s capsule rupture in their biopsy samples with an extensive rupture (52.8% of all glomeruli per sample). The markedly higher prevalence and severity of Bowman’s capsule rupture in anti-GBM disease, compared to other glomerulonephritis reported previously including ANCA-associated vasculitis (13), lupus nephritis (14), and IgA nephropathy (15), may reflect the exceptionally aggressive inflammation in the glomeruli and consequently poor prognosis in this disease.

Crescentic glomerulonephritis comprises a spectrum of kidney disorders distinguished by rapid deterioration of kidney function and the histological hallmark of glomerular crescent formation, with anti-GBM disease representing the most severe form (16). Glomerular crescents are composed of fibrin, proliferating PECs, T cells, and macrophages. While the precise mechanism underlying crescent formation remains incompletely understood, current evidence suggests that Bowman’s capsule rupture plays a crucial role in its progression (12, 17–19). The first hit of crescent formation, also well documented, is the formation of glomerular immune complex either *in situ* or from the circulation that induces podocyte injury and PEC activation, often resulting in early glomerular crescent formation. The second hit was recently proposed such that T cells and macrophages surrounding the glomerulus can migrate into the urinary space following breaches in Bowman’s capsule, promoting crescent formation (18). Our current study also revealed that the severity of Bowman’s capsule rupture positively correlated with crescent proportion, particularly the cellular–fibrous crescents, in patients with anti-GBM disease. This suggests that Bowman’s capsule rupture may occur in the late stage of crescent formation, supporting the notion that the “outside-in” attack of inflammation cells is a driver of crescent progression.

We firstly explored the clinical and prognostic significance of Bowman’s capsule rupture in patients with anti-GBM disease in this study. The severity of Bowman’s capsule rupture showed a strong correlation with kidney function, demonstrating a graded association with the incidence of oligoanuria, eGFR, and serum creatinine on diagnosis. Antibody-mediated autoimmunity has been considered as the major cause of anti-GBM disease (20–22). Our current findings demonstrated that the levels of anti-GBM antibody increased in parallel with Bowman’s capsule rupture percentage, suggesting that Bowman’s capsule rupture might contribute to the pathogenesis of anti-GBM disease. In terms of prognosis, our results showed that Bowman’s capsule rupture, as a single and simple pathological parameter on kidney biopsy, has discriminative ability to predict outcomes for both kidney survival and kidney recovery in patients with anti-GBM disease. Moreover, the incorporation of Bowman’s capsule rupture could enhance the predictive performance of two previously reported prognostic models—Floyd’s class and RRS model (3, 4). A similar predictive value of Bowman’s capsule rupture has been observed in patients with ANCA-associated vasculitis (13), suggesting its broader clinical relevance in crescentic glomerulonephritis.

Our study has limitations inherent to its retrospective design and small sample size due to disease rarity. Future multi-center studies with larger cohorts would be necessary to validate the role and characteristics of Bowman’s capsule rupture in anti-GBM disease.

In conclusion, Bowman’s capsule rupture is a distinct histopathological feature and a critical indicator of kidney injury in patients with anti-GBM disease. More importantly, this simple parameter alone demonstrates a strong predictive value for the kidney outcomes of patients. Routine quantitative assessment of Bowman’s capsule rupture percentage on kidney biopsies may offer potential benefits for its management.

## Data Availability

The original contributions presented in the study are included in the article/**Supplementary Material**. Further inquiries can be directed to the corresponding author.

## Supplementary material

Supplementary Figure 1 Decision tree analysis of Bowman’s capsule rupture (BCR) to predict kidney survival. Each box includes the number of patients who progressed into end-stage kidney disease (left)/the total number of patients.

Supplementary Figure 2 Decision tree analysis of Bowman’s capsule rupture (BCR) to predict kidney recovery. Each box includes the number of patients obtaining kidney recovery (left)/the number of patients receiving initial renal replacement therapy (right) with the median time of kidney recovery (below).
