# Supplementary figures and images for: Bowman’s capsule rupture and its clinical significance in patients with anti-glomerular basement membrane disease
Source: Front Immunol. 2025 Dec 4;16:1655319. doi: 10.3389/fimmu.2025.1655319 (PMC12711854; doi:10.3389/fimmu.2025.1655319)

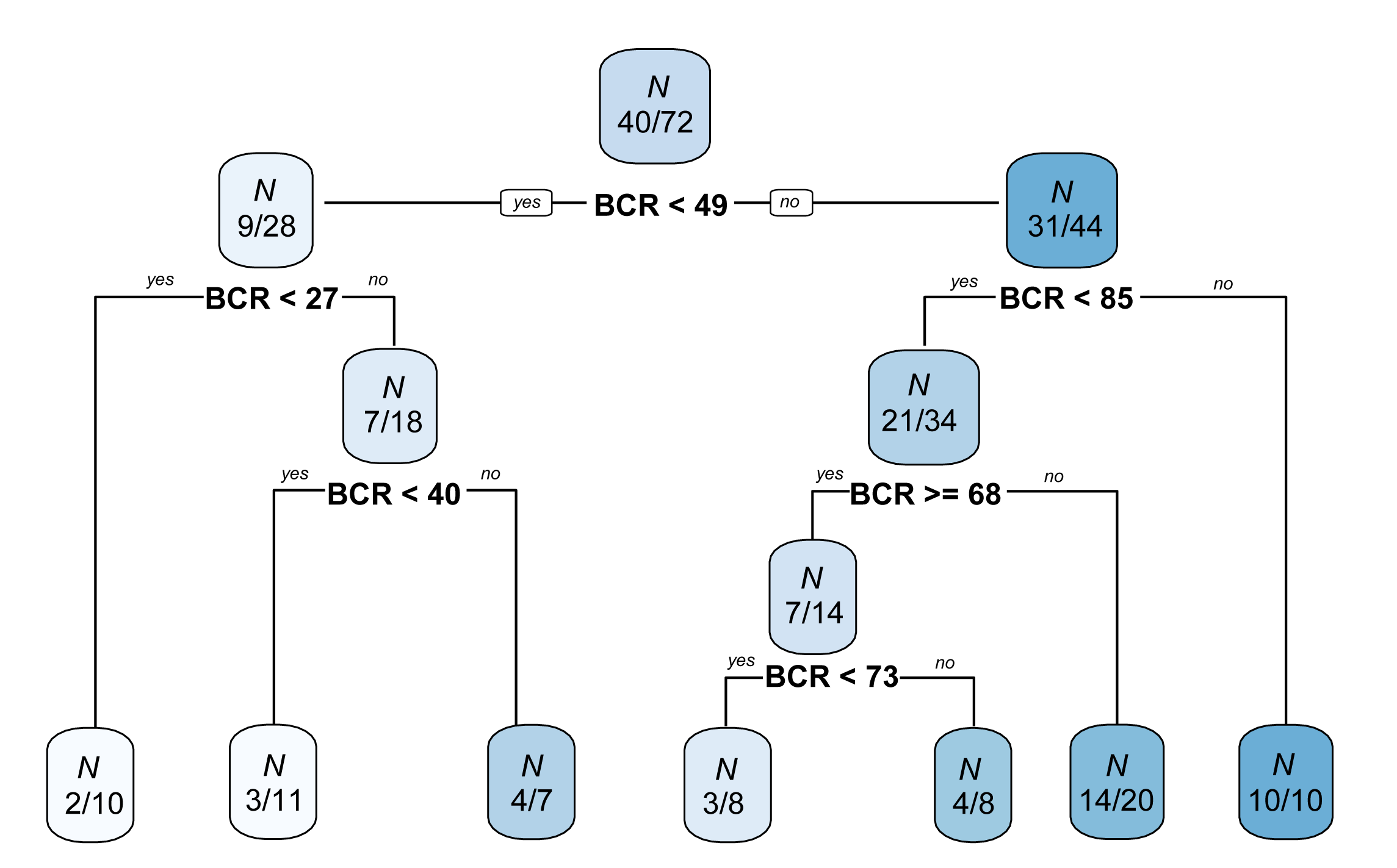

Supplement: Supplementary Figure 1 — Decision tree analysis of Bowman’s capsule rupture (BCR) to predict kidney survival. Each box includes the number of patients who progressed into end-stage kidney disease (left)/the total number of patients. [file Image1.tif]

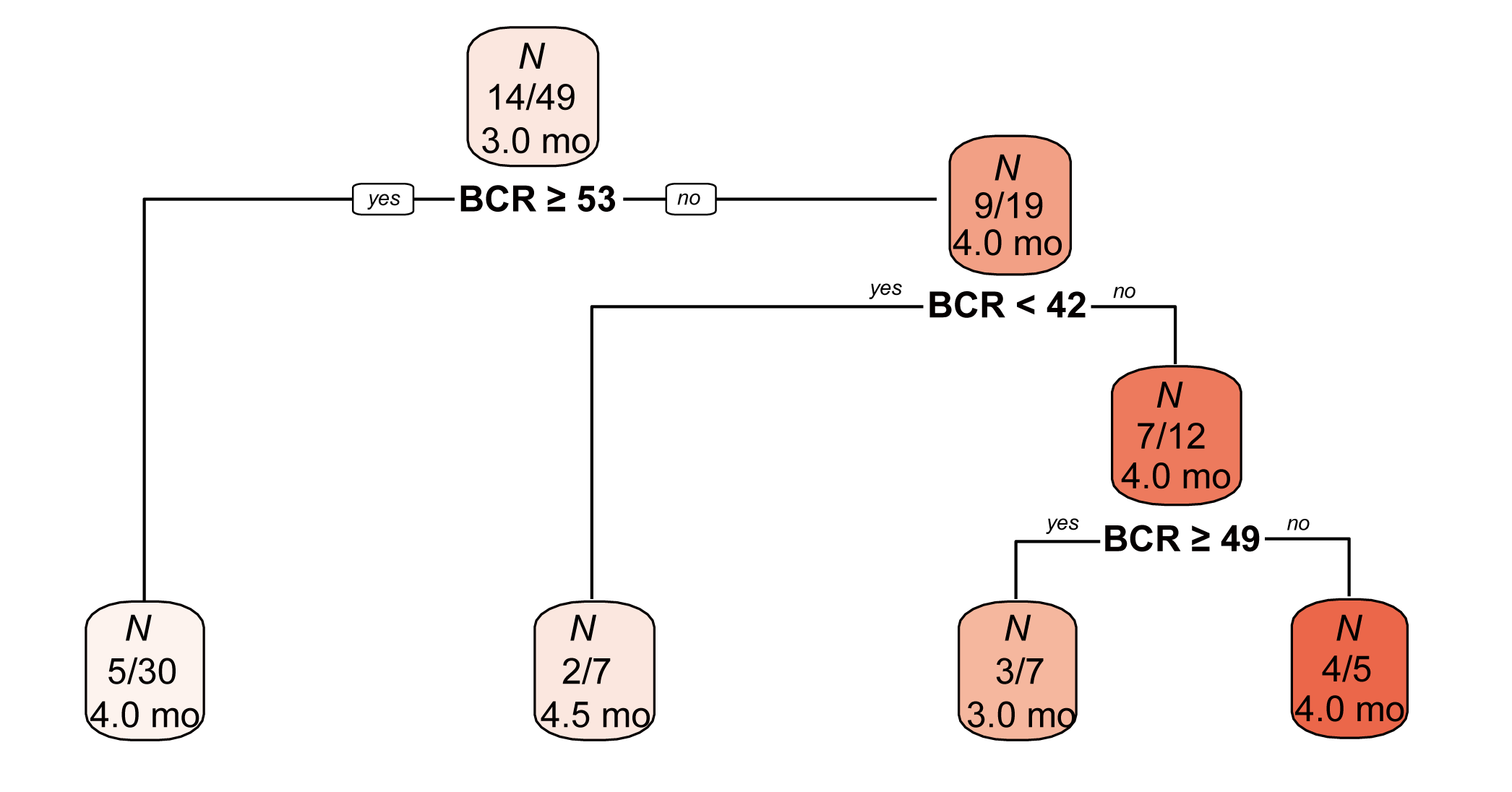

Supplement: Supplementary Figure 2 — Decision tree analysis of Bowman’s capsule rupture (BCR) to predict kidney recovery. Each box includes the number of patients obtaining kidney recovery (left)/the number of patients receiving initial renal replacement therapy (right) with the median time of kidney recovery (below). [file Image2.tif]
